# From pericarditis to giant cell arteritis: leveraging FDG PET CT for accurate diagnosis and treatment

**DOI:** 10.1093/rap/rkaf019

**Published:** 2025-02-19

**Authors:** Fatima K Alduraibi

**Affiliations:** Division of Clinical Immunology and Rheumatology, Department of Medicine, Beth Israel Deaconess Medical Center, Harvard Teaching Hospital, Boston, MA, USA; Division of Clinical Immunology and Rheumatology, Department of Medicine, University of Alabama at Birmingham, Birmingham, AL, USA; Division of Clinical Immunology and Rheumatology, Department of Medicine, King Faisal Specialist Hospital and Research Center, Riyadh, Saudi Arabia

An 81-year-old female with a past medical history of atrial fibrillation and sick sinus syndrome presented with persistent chest pain, low-grade fever and malaise. Initial workup revealed elevated CRP (204), ESR (41) and a moderate pericardial effusion without vegetation on echocardiogram. She was diagnosed with idiopathic pericarditis and treated with ibuprofen and colchicine.

She returned with recurrent fever, persistently high CRP (244), ESR (56) and leucocytosis. Haematologic, autoimmune and infectious studies were unremarkable. Repeat echocardiogram showed a small pericardial effusion, while cardiac MRA ruled out vasculitis. Axial enhanced chest CT revealed circumferential aortic wall thickening with enhancement and mild fat stranding (Fig. 1a, arrows), pericardial wall thickening with mild enhancement (Fig. 1b, arrowhead) and pericardial effusion (Fig. 1b, arrow). Fused coronal FDG PET/CT (Fig. 1c) and fused axial FDG PET/CT (Fig. 1d) demonstrated increased FDG uptake in the walls of the ascending aorta ( arrow), descending aorta and bilateral iliac arteries, confirming vasculitis consistent with giant cell arteritis. An axial non-enhanced chest CT (Fig. 1e) showed circumferential aortic wall thickening with mild fat stranding ( arrow).

**Figure 1.**
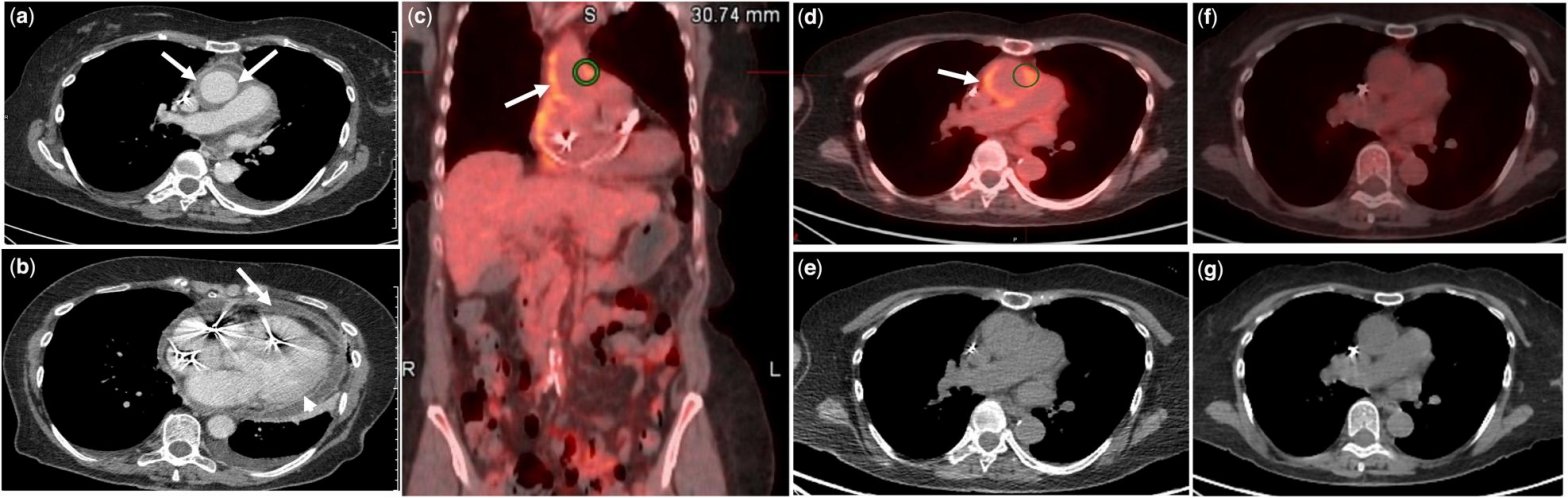
Whole-body FDG PET/CT showing pericarditis and vasculitis at baseline and resolution after 4 months of follow-up. (**a**) Axial enhanced chest CT scan, which revealed circumferential aortic wall thickening with enhancement and mild fat stranding (arrows). (**b**) Axial enhanced chest CT scan, which revealed pericardial wall thickening with mild enhancement (arrowhead), which indicates pericarditis and pericardial effusion (arrow). (**c**) Fused coronal FDG PET/CT scan, demonstrated an increased FDG uptake over the wall of the ascending aorta and pericardial wall (arrow). (**d**) Fused axial FDG PET/CT scan demonstrated an increased FDG uptake over the wall of the ascending (arrow). (**e**) Axial non-enhanced chest CT scan, which revealed circumferential aortic wall thickening with mild fat stranding (arrow). (**f**) Four-month follow-up. Fused axial FDG PET/CT scan demonstrated normal FDG distribution with no abnormal uptake. (**g**) Four-month follow-up axial non enhanced CT scan shows no abnormality

She was started on prednisone, leading to clinical improvement. A 4-month follow-up FDG PET/CT showed normal FDG distribution and resolution of the prior findings (Fig. 1f–g).

## Data Availability

The data underlying this article will be shared on reasonable request to the corresponding author.

